# Microbial profiling of dental plaque from mechanically ventilated patients

**DOI:** 10.1099/jmm.0.000212

**Published:** 2016-02-01

**Authors:** Kirsty M. Sands, Joshua A. Twigg, Michael A. O. Lewis, Matt P. Wise, Julian R. Marchesi, Ann Smith, Melanie J. Wilson, David W. Williams

**Affiliations:** ^1^​ Oral and Biomedical Sciences, School of Dentistry, Cardiff University, UK; ^2^​ Adult Critical Care, University Hospital of Wales, Heath Park, Cardiff, UK; ^3^​ School of Biosciences, Main Building, Park Place, Cardiff University, Cardiff, Wales, UK; ^4^​ Centre for Digestive and Gut Health, Imperial College London, London, UK

## Abstract

Micro-organisms isolated from the oral cavity may translocate to the lower airways during mechanical ventilation (MV) leading to ventilator-associated pneumonia (VAP). Changes within the dental plaque microbiome during MV have been documented previously, primarily using culture-based techniques. The aim of this study was to use community profiling by high throughput sequencing to comprehensively analyse suggested microbial changes within dental plaque during MV. Bacterial 16S rDNA gene sequences were obtained from 38 samples of dental plaque sampled from 13 mechanically ventilated patients and sequenced using the Illumina platform. Sequences were processed using Mothur, applying a 97 % gene similarity cut-off for bacterial species level identifications. A significant ‘microbial shift’ occurred in the microbial community of dental plaque during MV for nine out of 13 patients. Following extubation, or removal of the endotracheal tube that facilitates ventilation, sampling revealed a decrease in the relative abundance of potential respiratory pathogens and a compositional change towards a more predominantly (in terms of abundance) oral microbiota including *Prevotella* spp., and streptococci. The results highlight the need to better understand microbial shifts in the oral microbiome in the development of strategies to reduce VAP, and may have implications for the development of other forms of pneumonia such as community-acquired infection.

## Introduction

Mapping the oral microbiome has been a developing task for molecular microbiologists over the past decade (Wade, 2013a). Since the introduction of Sanger sequencing, technological evolution has allowed in-depth analysis of whole microbial communities and of individual species genomes (Lazarevic *et al.*, 2009; Perkins *et al.*, 2010). The oral cavity is colonized with an array of micro-organisms including bacteria, fungi and viruses, and currently over 800 bacterial species have been identified via culture-independent approaches (Avila *et al.*, 2009; Dewhirst *et al.*, 2010; Liu *et al.*, 2012; Wade, 2013b; Wang *et al.*, 2013; Zaura, 2012). The use of contemporary DNA-based technologies facilitates identification of uncultured, dormant and dying cells that are unable to replicate even on the most tailored media. In this way, the diversity of microbial species within the oral cavity of healthy individuals is increasingly well characterized. The microbiome of a range of oral niches has also been analysed, including the mucosal membranes, the cheeks and tongue and scrapings of dental plaque (Dewhirst *et al.*, 2010; Liu *et al.*, 2012). Several species within the polymicrobial biofilm of dental plaque have been associated with oral diseases including periodontitis and dental caries, and are increasingly linked with systemic infections and disorders. Analysis of dental plaque from patients with a particular illness, condition or hospitalized state could provide insight into the involvement of the oral microbiota in such systemic diseases (Gomes-Filho et al., 2010).

Although there are many shared species, different sites within the oral cavity have a characteristic and often unique microbial composition, relating to the different biological and physical properties of each site (Dewhirst *et al.*, 2010; Xu *et al.*, 2015). There is a large surface area provided by teeth for colonization and maturation of plaque. Areas with lowered oxygen potentials, such as the interproximal spaces of teeth and in deep periodontal pockets, are frequently colonized by large numbers of predominantly anaerobic species (Jefferson, 2004; Faran Ali & Tanwir, 2012). The difference in the microbial composition at different sites emphasizes the adaptability of the microbiome to specific surroundings. There is a natural progression in plaque development to maturation, with initial colonization by ‘pioneer’ bacteria including *Streptococcus* and *Lactobacillus* species, and subsequently combinations of anaerobic species such as *Prevotella*, *Veillonella* and *Porphyromonas* are detected (Kolenbrander *et al.*, 2005; Peyyala & Ebersole, 2013; Xie *et al.*, 2010).

The majority of critically ill patients require mechanical ventilation (MV) to facilitate survival. An endotracheal tube provides the interface between the patient and the ventilator and following insertion of the endotracheal tube (ETT; intubation) alterations can occur in the oral microenvironment and oral microbiome. These have been hypothesized to facilitate the colonization and proliferation of both respiratory and other potentially exploitative pathogens in oral and pulmonary niches (Berry *et al.*, 2011; Perkins *et al.*, 2010; Scannapieco *et al.*, 1992; Zuanazzi *et al.*, 2010). The mechanisms underlying this ‘microbial shift’ are not clear, but may, in part, be due to the physical presence of the ETT, which affects plaque clearance, saliva flow and mucosal drying, in addition to the interventions and medications related to the management of the underlying condition during critical illness.

Given the limitations of traditional microbiological culture, the aim of this current study was to use molecular community profiling to comprehensively analyse, on a longitudinal basis, the microbiome of dental plaque in patients undergoing MV. The use of high-throughput sequencing platforms in this way allows the investigation of a microbiome without placing specific focus or bias towards certain genera of pathogens and provides a more representative profile of the community.

## Methods

### Clinical study criteria and patient demographics

Ethical approval was obtained from National Research Ethics Service (NRES) within the Research Ethics Committee (REC), Wales (Ref: 13/WA/0039). Mechanically ventilated patients at a single University Hospital critical care unit were eligible for inclusion in the study if they were aged >18 years, had >8 original teeth and an expected survival of >24 h. Informed consent for participation in the study was obtained from the next of kin and patients reconsented if they regained capacity following recovery from critical illness. The number of decayed, missing and filled teeth (DMFT score) was used as an indication of previous general health status and oral hygiene (Becker *et al.*, 2007) and was determined by a dental practitioner. Standard oral care was performed following a critical care mouth assessment to determine level and frequency (every 6–12 h oral care) of oral care required. Ventilator-associated pneumonia (VAP) was diagnosed using the existing clinical pulmonary infection score (CPIS) score (with a score >6), with aetiology confirmed by blood cultures and quantitative microbiological culture (>10^3^ c.f.u. ml^− 1^) of the lower airways samples [bronchoalveolar lavage (BAL)/non-directed bronchoalveolar lavage (NBL)] (Estella & Álvarez-Lerma, 2011; Hellyer *et al.*, 2015; Kalanuria *et al.*, 2014; Pugin *et al.*, 1991; Zilberberg & Shorr, 2010). Antibiotics were prescribed at clinicians’ discretion and further stewardship was provided by a three-times-weekly wardround with clinical microbiologists.

### Samples for bacterial community profiling

A total of 38 samples of dental plaque were collected from 13 mechanically ventilated patients using paper points (size 40, QED) for Illumina sequencing (Research and Testing Laboratory). Dental plaque samples were collated according to the time of MV and, when possible, grouped into the following categories: ETT intubation (start), midpoint, end and/or admission to ward. Dental plaque was collected using paper points (size 40, QED), with a total of six paper points used per sample (three supragingival, three subgingival) and stored in transport medium (TM). Both supragingival and subgingival dental plaque samples were collected and pooled. The composition of dental plaque can differ between these two sites, and such, pooled dental plaque was used to represent the entire community. The TM composition was as described by Syed & Loesche (1972).

### DNA extraction from dental plaque samples stored at 4 °C

Total bacterial DNA from subgingival and supragingival dental plaque pooled for a single patient was extracted using a Qiagen kit (DNA extraction kit Yeast/Bac). All incubation steps were extended to 1 h to maximize DNA elution. To efficiently lyse the bacteria, resuspended plaque in lysis solution was transferred to a pathogen-lysis tube (Qiagen) for a 1 min bead-beating step (shaking speed 3450 oscillations min^− 1^; Mini-bead beater, Biospec products).

### Gel electrophoresis with 16S rRNA gene PCR bacterial primers

To confirm the presence of DNA and visualize the amplicons before the sample was submitted for Illumina sequencing, the extracted DNA was subjected to PCR using the primer pair of 27f (GTGCTGCAGAGAGTTTGATCCTGGCTCAG) and 1492r (CACGGATCCTACGGGTACCTTGTTACGACTT) (Eurofins MWG Operon) to amplify bacterial 16S rRNA genes (Dalwai *et al.*, 2007; Zuanazzi *et al.*, 2010). PCR thermal cycling parameters consisted of an initial denaturation step of 95 °C for 1 min, followed by 26 cycles of 94 °C for 45 s, 50 °C for 45 s and 72 °C for 90 s (Thermocycler, G-Storm). A final single cycle extension step of 72 °C for 15 min was also included.

### Species-specific PCR of *Pseudomonas aeruginosa* from pooled dental plaque

Extracted DNA from dental plaque samples with culture-positive *Pseudomonas aeruginosa* isolation was subject to species-specific PCR targeting the *ecfX* gene using the primers ECF1 (5′-ATGGATGAGCGCTTCCGTG-3′), and ECF2 (5′-TCATCCTTCGCCTCCCTG-3′) (Lavenir *et al.*, 2007). PCR cycling conditions were as follows: there was an initial denaturation step of 95 °C followed by 35 cycles of 94 °C for 45 s, 58.4 °C for 45 s and 72 °C for 1 min ending with 5 min at 72 °C.

### Preparation for MiSeq sequencing

Amplicons were stabilized in a DNA elution reagent (Qiagen). Amplicon sequencing using the Illumina MiSeq bacterial primers (28F: GAGTTTGATCNTGGCTCAG and 388R: TGCTGCCTCCCGTAGGAGT) was performed by Research and Testing Laboratory (Austin, Texas, USA) to generate multiple sequences of 250 bp overlapping at the V4 region of the 16S rRNA gene.

### Phylogenetic identification and data analysis

Data analysis from the raw sequences generated from the Illumina platform was performed using Mothur (Schloss *et al.*, 2009) and used to quality check, pre-process, align and join sequences to obtain a total number of sequences in each sample. Sequences were scanned for errors by a series of error command checks. To analyse the microbial communities of samples, Mothur was used to cluster the data according to operational taxonomic units (OTUs), to a species level of 97 % similarity (sequence data < 97 % confirmation were not identified in this study). Singletons and any OTUs which were not found more than 10 times in any sample were collated into OTU_singletons and OTU_rare phylotypes, respectively, to maintain normalization and to minimize artefacts. The raw data output from Mothur (phylogenetic data) was analysed using a combination of statistical programs R-script (R Development Core Team 2008), stamp (Parks & Beiko, 2010), SPSS V20 and Microsoft Excel. The two methods used in this study to compare the similarity between each sample were Jaccard's index of similarity and the Bray–Curtis distance measure (measure of dissimilarity) (Lozupone & Knight, 2005; Wang *et al.*, 2013). The terms ‘non-oral’ and ‘potential respiratory pathogens’ were employed to describe those micro-organisms not generally isolated from or considered part of the oral microbiome. Weighted Unifrac distance matrices were analysed in R using non-metric multidimensional scaling ordination and the shared OTU file was used to determine the number of times that an OTU was observed in multiple samples and for multivariate analysis in R. OTU taxonomies (from phylum to genus) were determined using the RDP MultiClassifier script to generate the RDP taxonomy (Wang *et al.*, 2007) while species level taxonomies of the OTUs were determined using the USEARCH algorithm combined with the cultured representatives from the RDP database (Edgar, 2010). Alpha and beta indices were calculated from these datasets with Mothur and R using the Vegan package.

## Results

### Sequencing data details and patient demographics

In total, 1 911 760 sequence reads (before quality control) were determined from 38 samples of pooled supragingival and subgingival dental plaque from 13 mechanically ventilated patients. OTUs were subsampled to the lowest read count of 1016, which retained 97 % of all OTU counts; and the mean and median read lengths were 310 and 339 bp, respectively. The criteria for patients to be included in this study were as follows: >18 years old, >8 teeth, >24 h life expectancy, and anticipated duration of MV >24 h. The patient demographics are summarized in Table 1. A total of eight male and five female subjects with an age range between 18 and 75 years were included. The DMFT scores for 10/13 patients were >10. A total of 6/13 patients were diagnosed with VAP. An additional two patients were admitted with pre-existing respiratory disorders, as shown in Table 1. Out of a total of nine patients exhibiting potential respiratory pathogens within their dental plaque community during ETT intubation, five were treated with antibiotics >48 h after ETT intubation, and three patients were treated with antibiotics from the beginning of ETT intubation.

**Table 1:** Patient demographics including age, gender, admission details, DMFT score, incidence of a microbial shift and VAP

**Patient**	**Age**	**Gender**	**Admission**	**DMFT score**	**Microbial shift**	**VAP**
1	33	M	Left main bronchus clot	4	Yes	Yes
2	55	F	Subarachnoid haemorrhage	19	No	Yes
3	54	F	Respiratory failure	11	Yes	No
4	39	M	Asphyxia (hanging)	–	No	No
5	69	M	Poly-trauma, haemopneumothorax, dissected aorta	22	Yes	Yes
6	72	M	Heart attack	12	Yes	No
7	68	M	Community-acquired pneumonia	13	No	No
8	18	F	Encephalopathy	0	No	Yes
9	58	F	Encephalopathy, cerebral oedema	19	Yes	No
10	67	M	Hypoxia, seizures, bronchial haemorrhage	11	Yes	Yes
11	48	M	Heart attack, head trauma	12	Yes	Yes
12	75	F	Laparotomy for small bowel obstruction and faecal peritonitis	16	Yes	No
13	39	M	Spontaneous intracranial haemorrhage	11	Yes	No

### OTU analysis of dental plaque collected from MV patients

**Fig. 1:**
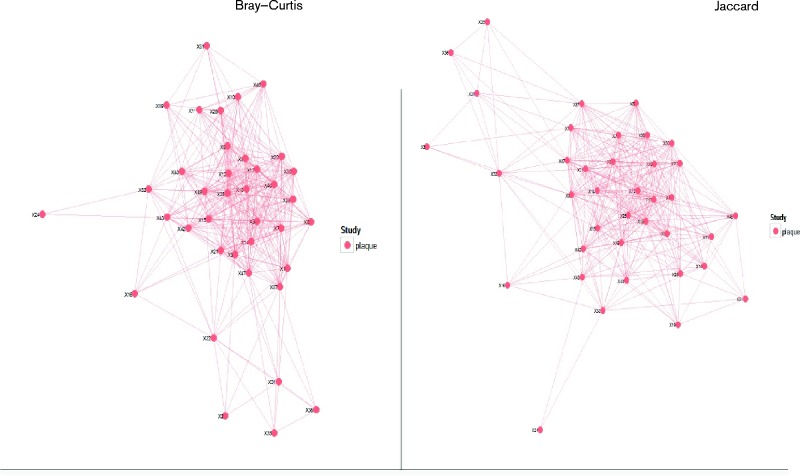
Bray–Curtis and Jaccard analyses of dental plaque samples. A red spot represents each sample; the sample number follows X. By both methods, 31 samples are seen to cluster tightly, with seven outliers identifiable.

Clustering analysis was performed to provide an overview of the level of similarity within all dental plaque samples. Fig. 1 shows the clustering results using Jaccard's index of similarity and the Bray–Curtis distance methods of analysis. The figures show that the majority of samples form a tight cluster (multiple overlapping lines connecting samples) with a minority of samples falling outside the tightly clustered group. Analysis of the data generated from pooled dental plaque samples was carried out using the three following criteria: prevalence of non-oral pathogens, the most abundant species during MV and, finally, the grouping of dental plaque samples to four time points.

**Fig. 2:**
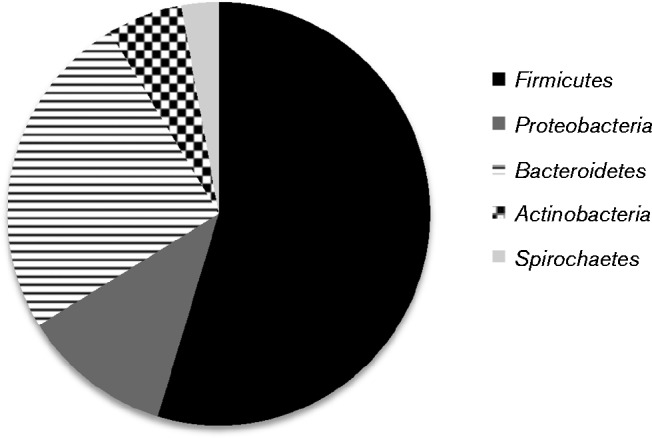
The proportion of each of the major phyla identified in dental plaque samples.

A total of five bacterial phyla were represented during this study of 13 patients (Fig. 2). Over 50 % of all organisms identified belonged to the *Firmicutes* phyla. Furthermore, over 100 bacterial species belonging to a total of 40 different genera were identified. Of the 40 genera identified within this group of patients, six represented species not generally considered to be usual members of the dental plaque community, including *Enterococcus* and *Staphylococcus* species. The four most frequently occurring microbial species within the 38 dental plaque samples were *Staphylococcus aureus* present in 68 %, *Streptococcus pseudopneumoniae* (66 %), *Enterococcus faecalis* (37 %) and *Escherichia coli/Shigella flexneri* (32 %). These microbial species were the four most commonly isolated within the 38 samples of dental plaque, but not necessarily the most abundant.

### Microbial changes within the community of dental plaque during MV

**Fig. 3:**
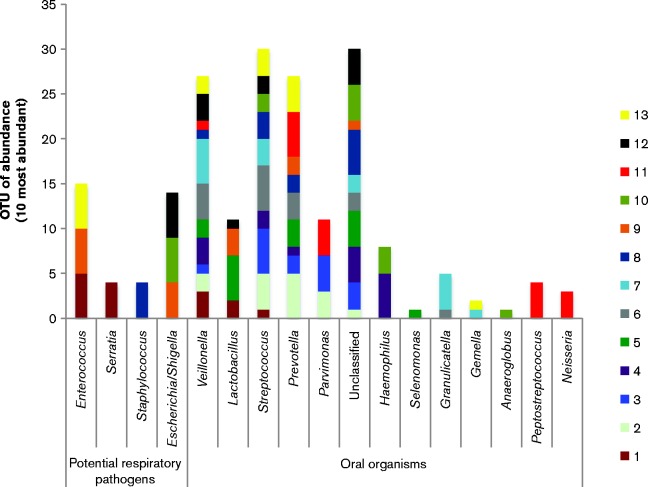
A stacked bar graph representing the five most abundant organisms present in the dental plaque community at the start of mechanical ventilation for each of the 13 patients. Each patient is represented by a different colour.

To determine whether ‘microbial shifts’ occurred during MV, and to ascertain the extent thereof, the dental plaque community at ETT-insertion (time point 1) was analysed and compared for each patient with that at subsequent time points. In the majority of cases, sample 1 was collected shortly after ( < 12 h) intubation to reflect the dental plaque community at the time of initiation of MV. The number of identified OTU sequences was used as a direct measure of species abundance. Fig. 3 shows the five most abundant microbial species within dental plaque at the first time point (insertion of ETT, introduction of MV) comparing prevalence of oral species and potential respiratory pathogens. Both potential respiratory pathogens and oral micro-organisms are represented. The stacked bar graph compares the five most abundant species per patient (with the largest bar representing the most abundant species). The occurrence of multiple bands in oral commensal genera such as *Streptococcus*, *Veillonella* and *Prevotella* indicated their presence in multiple patients. A total of seven patients were colonized with potential respiratory pathogens from within 12 h of the onset of MV. When present, potential pathogens were amongst the most predominant micro-organisms as illustrated by larger bars on the graph.

Overall, analysing a proportion of the most abundant species in the plaque of each patient highlights a large degree of variability in bacterial composition of the dental plaque with large numbers of respiratory pathogens isolated at early stage intubation (7 out of the 13 patients profiled during MV).

**Fig. 4:**
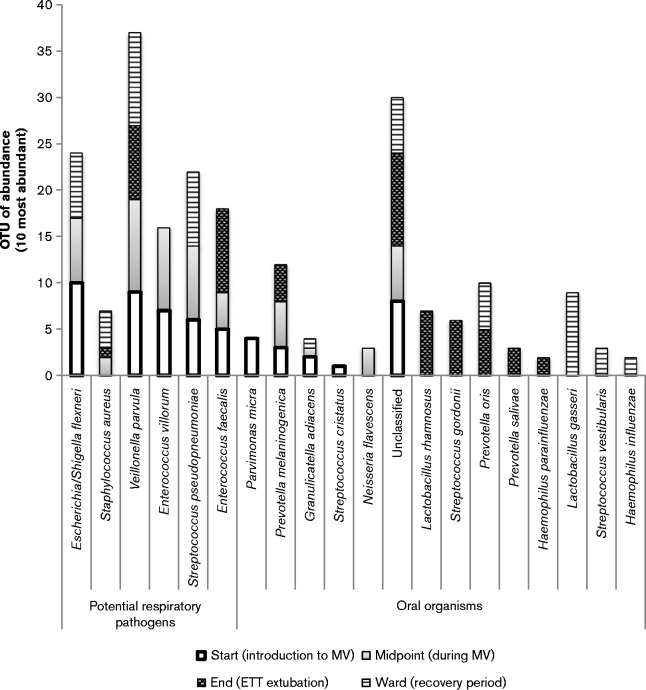
A stacked bar graph representing the 10 most abundant microbial species within dental plaque, at each of the four time points of MV and recovery. This bar graph allows the comparison between oral organisms and putative respiratory pathogens.

**Fig. 5:**
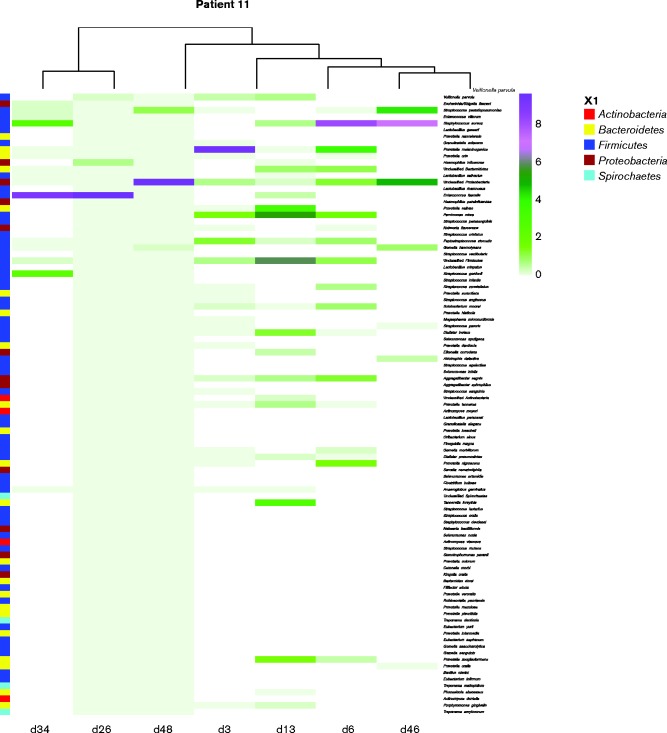
Heat map indicating the level of phyla, species variation and species abundance during MV.

**Fig. 6:**
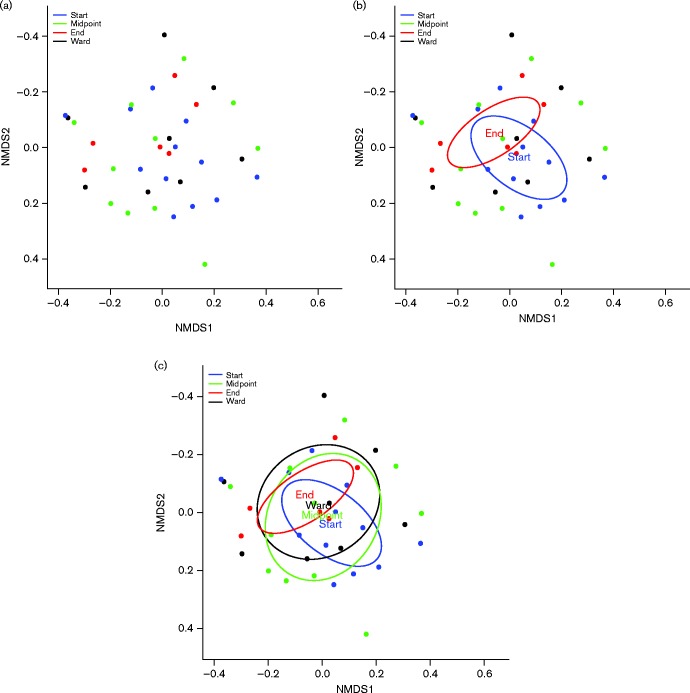
Non-metric multidimensional scaling (NMDS) analysis of grouped dental plaque samples. (a) Scatter representations of dental plaque communities and (b) the grouping, and therefore relationship between dental plaque communities from the start of intubation and at the end of extubation. (c) Overlap of communities at all sample time points.

Further analysis of the 10 most abundant species at each sampling time point relating to MV highlights that longitudinal changes in the composition of the dental plaque occurred. As shown in Fig. 4, dental plaque samples were grouped according to stage of MV for comparative analysis revealing that a shift in the composition of dental plaque was evident between the stages of MV. Dental plaque samples obtained at the start and midpoint of MV showed a high prevalence of bacteria not frequently identified in the oral cavity, also demonstrated in the heat map (Fig. 5). *Veillonella* are regarded as frequent members and a key component of dental plaque, and this was confirmed by the present study with *Veillonella* species present within the top three most abundant microbial species during the entirety of MV and into the recovery period. *Escherichia coli/Shigella flexneri* and *Staphylococcus aureus* persisted as abundant members of the dental plaque biofilm into the recovery period. Non-metric multidimensional scaling (NMDS) coupled with *t*-tests were used to analyse the level of similarity between sample types using non-parametric relationships (Fig. 6). Each sample was furthermore assigned coordinates based on weighted Unifrac (phylogenetic community distance measure) analysis to generate a scatter plot. Although there were overlapping components, statistically the communities were shown to change during MV. Pairwise comparisons using *t*-tests with pooled sd were performed to compare each of the time point groups. *P* value adjustment was performed using the FDR Benjamini–Hochberg (BH) method, to control the false discovery rate when performing multiple comparisons. The parameters used were Unifrac against time, to determine whether time point communities were statistically different. The differences in the plaque compositions at the start and midpoint were shown to be statistically significant, *P* = 0.0033. Furthermore, the differences in the communities between the start and end (ETT-extubation sampling) were also significantly different (*P* = 0.0403), with a *P* value of *P* = 6.3 × 10^− 5^ when comparing the communities between midpoint and end of MV.

In addition to significant species changes and the introduction of many non-oral pathogens during MV, the relative abundance of respiratory pathogens was shown to change during MV (Table 2, Figs 4, 5 and 6). Table 2 shows the relative variation in abundance for all non-oral bacteria found within the dental plaque during ventilation. Inclusion of non-oral pathogens to the plaque community was demonstrated for all 13 patients. However, for four patients these micro-organisms were detected only in low numbers. The dental plaque microbiome for the remaining nine patients was shown to change to include relatively increased numbers of non-oral pathogens, with eight patients showing notable increases in the abundance of potential respiratory pathogens during MV.

**Table 2:** Abundance measurements of putative respiratory pathogens (total OTUs) A total of nine out of 13 patients had >2 respiratory pathogens during MV; d, day during mechanical ventilation; ward, collection of dental plaque on the ward (post ETT extubation).

**Potential respiratory pathogen**	**Total OTUs isolated**				
**Patient 1**	**d1**	**d4**	**d5**		
*Enterococcus villorum*	623	983	135		
*Staphylococcus aureus*	11	2	35		
*Enterococcus faecalis*	0	1	20		
*Serratia nematophila*	214	0	30		
**Patient 3**	**d1**	**d11**	**ward**		
*Serratia nematophila*	1	0	0		
*Streptococcus pseudopneumoniae*	155	766	796		
*Staphylococcus aureus*	1	241	217		
**Patient 5**	**d1**	**ward**			
*Streptococcus pseudopneumoniae*	11	1			
*Staphylococcus aureus*	0	3			
*Enterococcus faecalis*	4	0			
**Patient 6**	**d1**	**d6**	**ward**		
*Streptococcus pseudopneumoniae*	159	126	7		
*Staphylococcus aureus*	2	1	0		
*Enterococcus faecalis*	0	4	0		
**Patient 9**	**d1**	**d5**	**ward**		
*Escherichia coli*	349	385	1001		
*Enterococcus villorum*	451	296	5		
*Staphylococcus aureus*	0	17	1		
*Enterococcus faecalis*	0	136	4		
**Patient 10**	**d1**	**d3**	**d6**		
*Escherichia coli*	949	863	13		
*Enterococcus villorum*	0	3	0		
*Staphylococcus aureus*	2	1	5		
*Enterococcus faecalis*	0	1	0		
**Patient 11**	**d3**	**d13**	**d26**	**d34**	**d48**
*Escherichia coli*	0	3	1	18	0
*Streptococcus pseudopneumoniae*	12	0	0	18	102
*Enterococcus villorum*	0	0	0	22	0
*Staphylococcus aureus*	0	27	9	23	4
*Enterococcus faecalis*	0	22	916	549	2
*Stenotrophomonas pavanii*	0	0	1	0	0
**Patient 12**	**d1**	**d8**			
*Escherichia coli*	422	0			
*Streptococcus pseudopneumoniae*	0	225			
*Staphylococcus aureus*	3	11			
*Enterococcus faecalis*	29	20			
*Clostridium bolteae*	20	0			
**Patient 13**	**d1**	**d27**	**ward**		
*Streptococcus pseudopneumoniae*	91	0	0		
*Staphylococcus aureus*	0	2	104		
*Enterococcus faecalis*	578	0	54		
*Stenotrophomonas pavanii*	0	0	2		

**Fig. 7:**
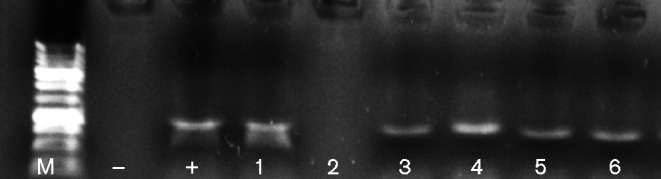
Species-specific PCR for the detection of *Pseudomonas aeruginosa* from dental plaque samples in cases where *Pseudomonas aeruginosa* was isolated during microbial culture. A total of five dental plaque samples were positive for *Pseudomonas aeruginosa*. M, size marker.

Although previous literature (Luyt *et al.*, 2014) and adjoining studies within our laboratories (Study Ref: 13/WA/0039) have isolated *Pseudomonas aeruginosa* by cultural methods within the dental plaque of mechanically ventilated patients, this species was not identified using the culture-independent approaches performed within this study. A total of six from 38 samples analysed within this study were microbial culture positive for *Pseudomonas aeruginosa*. Following species-specific PCR of these dental plaque samples, five out of six were positive for *Pseudomonas aeruginosa*, as shown in Fig. 7. This suggests that although *Pseudomonas aeruginosa* can be isolated from the dental plaque community using microbial culture, this result may be due to the fact that members of this genus are well adapted to grow on culture media and are over-represented when using culture-based approaches. Although significant numbers of respiratory pathogens were detected, culture-independent analysis did not detect any *Pseudomonas aeruginosa* from mechanically ventilated patients within this study.

### Analysis of dental plaque communities post ETT extubation

**Fig. 8:**
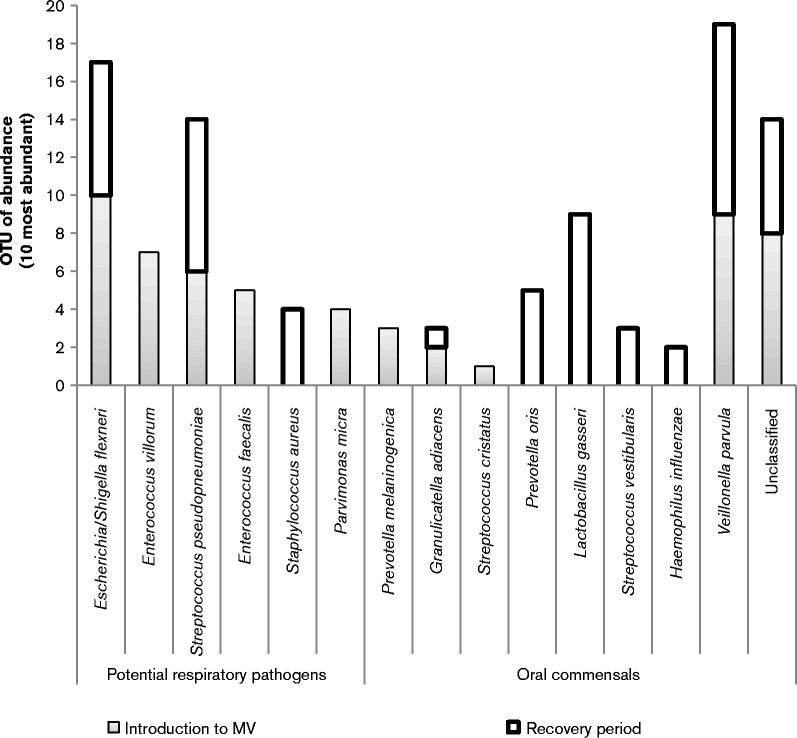
Stacked bar graph representing the 10 most abundant microbial species for the biofilm community time point for groups start and end. The two most abundant species within dental plaque sampled on the ward were oral organisms, *Veillonella* and *Lactobacillus.* A decrease in *Escherichia coli* and absence of *Enterococcus* species was also observed post extubation.

The final part of the study was to elucidate whether the communities of dental plaque at the beginning of intubation (start) were similar to those of samples collected post-extubation (ward) (Fig. 8). There were similarities between these two groups of dental plaque, initially suggesting that the microbial shift does not necessarily revert back to normal oral microbiota upon extubation. Furthermore, patients (patients 3, 9 and 13) were shown to be colonized with respiratory pathogens, namely *Escherichia coli/Shigella flexneri*, *Staphylococcus aureus* and/or *Streptococcus pseudopneumoniae*, in the post-ETT extubation period, as shown in Table 2. There was no significant difference between these groups. A pairwise *t*-test measuring the weighted Unifrac against the datasets indicated a lack of significance (*P* = 0.1945), furthermore highlighting the occurrence of both oral and respiratory organisms in both start- and ward-designated samples. However there was a noticeable decrease in the occurrence and abundance of potential respiratory pathogens, especially Gram-negative respiratory pathogens such as *Escherichia coli*. The dental plaque communities may not revert back to levels found in the initial samples; however, 50 % of the most abundant species in post-ETT samples were frequent members of the oral microbiota such as streptococci, lactobacilli and *Veillonella* species, with a decrease in respiratory pathogens. This can be seen in Fig. 8, by visually comparing the distribution of oral organisms and respiratory pathogens. *Staphylococcus aureus* was present only in low numbers in mechanically ventilated patients at the start of MV; however, this species was detected during MV (Fig. 3) and during the recovery period (Fig. 8).

## Discussion

To our knowledge, this is the first time that molecular community profiling has been used to characterize the changes that occur in dental plaque during and after MV. This study examined the dental plaque microbiome at multiple stages during MV and into the recovery period. An average of three dental plaque samples were collected per patient covering four different time points, with the patient demographics summarized in Table 2. Longitudinal analysis of the polymicrobial dental plaque community in this way revealed that there were significant differences in bacterial composition at discrete stages of MV. Next-generation sequencing-based applications such as community profiling allow simultaneous detection of micro-organisms and the generation of species quantitative data (Zaura, 2012). During this study, MiSeq Illumina sequencing provided comprehensive microbial profiles for mechanically ventilated patients and highlighted increases in relevant pathogens within dental plaque of ventilated patients. These findings confirmed and considerably extended those of culture-based studies that have previously demonstrated microbial shifts, which may facilitate the development of secondary respiratory infections such as VAP (Mori *et al.*, 2006; Zuanazzi *et al.*, 2010). In this context, it has been proposed that the placement of the ETT may actually facilitate a change in the biochemical composition of the oral cavity (Cairns *et al.*, 2011). The microbial changes demonstrated that several non-oral genera can co-exist in the dynamic, ever-changing biofilm that is dental plaque.

Interestingly, *Pseudomonas aeruginosa* was not identified within the dental plaque of mechanically ventilated patients using the culture-independent method within this study, but was identified in four out of 13 patients via microbial culture. Previous studies have suggested *Pseudomonas aeruginosa* to be a predominant colonizer of dental plaque during MV and a causative pathogen of VAP (Parker *et al.*, 2008; Raad *et al.*, 2011; Tarquinio *et al.*, 2014). Current microbiological analysis relies on cost effective, readily available and reproducible microbial culture. *Pseudomonas aeruginosa* is a rapid colonizer on nutritional media and can effectively outcompete the growth of other microbial species perhaps inaccurately presenting as a predominant species in this setting. The composition of dental plaque is extremely diverse with many species regarded as currently uncultivable, reinforcing the potential for *Pseudomonas aeruginosa* to out-compete and be over-represented on microbiological media. Although, as explained, *Pseudomonas aeruginosa* is considered an opportunistic pathogen of the oral cavity within mechanically ventilated patients, further high-throughput studies could elucidate the true extent of *Pseudomonas aeruginosa* within dental plaque during MV to determine whether *Pseudomonas aeruginosa* should still be considered the predominant bacterium in this context compared with *Streptococcus pneumoniae*, *Staphylococcus aureus* and Gram-negative species such as *Escherichia coli*.

Sachdev *et al.* (2013), and other groups have reported that the amount of dental plaque in hospitalized and ventilated patients increases (Jones *et al.*, 2011) with an accompanying increase in gingival inflammation. The latter is a marker of host immune activity in response to the elevated plaque levels and can highlight the importance of plaque scoring in critical care medicine (Wise & Williams, 2013). Oral hygiene, predominantly tooth brushing, can be effective in disrupting the plaque biofilm; however, small amounts of potentially pathogenic biomass firmly attached to the enamel surface are likely to remain (Alhazzani *et al.*, 2013).

Several organisms, predominantly Gram-negative bacilli, which may enter the oral cavity, have the ability to exploit early plaque colonizers such as streptococci which have attached to the surface of the tooth (Perkins *et al.*, 2010). Proteins within saliva aid the attachment of early pioneer organisms including streptococci and have a key role in oral microbial homeostasis (Hojo *et al.*, 2009). Therefore, any alteration in saliva production or composition may contribute to changes in microbial composition within dental plaque. Non-oral organisms, from several genera, are rapid formers of resistant biofilms (Fux *et al.*, 2005) and are relatively efficient in competing with resident members of the microbiome following changes in oral homeostasis, and may significantly affect the biofilm composition (Marsh, 2003, 2006). Furthermore, this may add to changes in the microenvironment of the oral cavity within intubated patients. The insertion of the ETT, coupled with the immunocompromised status of many critically ill patients, and administered antibiotics and drugs, may all contribute to the initial changes at the proteome level of saliva.

Dental plaque is known to be diverse and dynamic with microbial members responding and adapting to their microenvironment (Hojo *et al.*, 2009; Robinson *et al.*, 2006). Within this study, a total of 40 genera were identified, ranging from frequently identified and studied genera such as streptococci, to less obvious organisms such as *Tannerella* spp. and *Oscillibacter* spp., some of which lesser known genera have been revealed in similar community profiling studies (Galimanas *et al.*, 2014). *Haemophilus influenzae*, an organism associated with respiratory infection, was also isolated within the dental plaque of mechanically ventilated patients. Although an opportunistic pathogen, *Haemophilus influenzae* is a common commensal of the nasopharynx (King, 2012). Physical alterations (such as the insertion of ETTs) may influence microbial detachments and translocation within the upper and lower respiratory system. The dynamics and influence of dental plaque may in fact be underestimated and the biomass of dental plaque can be a significant contributing factor to several oral and systemic infections, such as VAP. The microbial shift occurred in a high proportion of mechanically ventilated patients, and at least one pathogen was isolated in the dental plaque of all 13 patients tested in this study. *Streptococcus pseudopneumoniae* has also been suggested as an early onset VAP causative pathogen, alongside *Staphylococcus aureus* and Gram-negative bacilli (Kalanuria *et al.*, 2014). *Enterococcus faecalis*, an opportunistic pathogen identified within the four most frequently occurring species in dental plaque during MV, has the ability to integrate into the polymicrobial dental oral biofilm *in situ* (Al-Ahmad *et al.*, 2010). *Enterococcus faecalis*, although not commonly regarded as a member of the oral microbiota in health, may be isolated from the oral cavity in some individuals and is interestingly frequently isolated from secondary endodontic infections (Al-Ahmad *et al.*, 2009).

Antimicrobial mouthwashes, such as chlorhexidine, used as a preventative strategy for VAP have generated conflicting reports within current literature, as to their efficacy in improving patient outcomes with regard to secondary infection (Bellissimo-Rodrigues *et al.*, 2009; Jones, 1997; Klompas *et al.*, 2014). Previous reports indicate that low concentrations of chlorhexidine may have effects largely against native oral biofilm species, particularly after initial plaque removal via brushing (Zanatta *et al.*, 2007). Other studies including a trial reported by Scannapieco *et al.* (2009) suggest activity of chlorhexidine against *Staphylococcus aureus*, and highlight the need to evaluate whether this and similar antimicrobials are effective against the biofilm consortia of intubated patients, which in this study are shown to be predominantly colonized by putative and opportunistic pathogens.

Dental plaque analysis post ETT extubation revealed a decrease in respiratory pathogens for many patients comprehensively analysed, and the community begins to revert back to that of predominantly oral organisms. However, potential pathogens were isolated from dental plaque obtained during patient recovery from critical illness, suggesting that once these organisms establish within the biofilm, they may be hard to eliminate completely. This may be related to the continued relatively immunocompromised state of the patient during the post critical illness phase (Frazier & Hall, 2008). Future studies could analyse the dental plaque microbiome of a larger patient sample size to statistically reflect the community of critical care patients at pre-defined time points during MV.

## Conclusion

This study employed molecular community profiling to characterize the plaque microbiome of patients during and after MV. A microbial shift in the composition of dental plaque was demonstrated with the incorporation of several potential respiratory pathogens including *Staphylococcus aureus*, *Streptococcus pseudopneumoniae* and *Escherichia coli*. Interestingly, and in contrast to previous cultural studies, *Pseudomonas aeruginosa* was not represented in the molecular analysis. Both the prevalence and abundance of potential respiratory pathogens were shown to decrease following extubation. However, some of these microbial species could still be identified at low levels within the community, and may therefore serve as a reservoir for infection in the longer term. To our knowledge this is the first report of the comprehensive characterization of dental plaque in this patient group. A better understanding of the compositional changes with dental plaque will usefully inform interventional strategies to reduce the incidence of VAP.
